# Clinical dynamic visual acuity in patients with cerebellar ataxia and vestibulopathy

**DOI:** 10.1371/journal.pone.0255299

**Published:** 2021-07-29

**Authors:** Michaela Dankova, Jaroslav Jerabek, Dylan J. Jester, Alena Zumrova, Jaroslava Paulasova Schwabova, Rudolf Cerny, Silvia Kmetonyova, Martin Vyhnalek

**Affiliations:** 1 Department of Neurology, Centre of Hereditary Ataxias, 2nd Faculty of Medicine, Charles University and Motol University Hospital, Prague, Czech Republic; 2 School of Aging Studies, University of South Florida, Tampa, FL, United States of America; 3 Department of Paediatric Neurology, 2nd Faculty of Medicine, Charles University and University Hospital Motol, Prague, Czech Republic; University of Rochester, UNITED STATES

## Abstract

Deterioration of dynamic visual acuity (DVA) as a result of impaired vestibulo-ocular reflex (VOR) has been well described in peripheral vestibulopathies, however, changes in DVA in patients with degenerative cerebellar ataxias (CA) and its relation to VOR impairment in these patients has not yet been evaluated. Our aim was to assess the alterations of DVA in CA and to evaluate its relation to vestibular function. 32 patients with CA and 3 control groups: 13 patients with unilateral and 13 with bilateral vestibulopathy and 21 age matched healthy volunteers were examined by clinical DVA test, VOR was assessed by video Head Impulse Test and caloric irrigation. The severity of ataxia in CA was assessed by Scale for the assessment and rating of ataxia (SARA). Relationship between DVA and vestibular function in CA patients was examined by linear regressions. DVA impairment was highly prevalent in CA patients (84%) and its severity did not differ between CA and bilateral vestibulopathy patients. The severity of DVA impairment in CA was linked mainly to VOR impairment and only marginally to the degree of ataxia. However, DVA impairment was present also in CA patients without significant vestibular lesion showing that central mechanisms such as impairment of central adaptation of VOR are involved. We suggest that the evaluation of DVA should be a standard part of clinical evaluation in patients with progressive CA, as this information can help to target vestibular and oculomotor rehabilitation.

## Introduction

Dynamic visual acuity (DVA) is an ability to maintain clear vision during head movement. DVA is mediated mainly by vestibulo-ocular reflex (VOR) which enables gaze stabilization during high-velocity head movements by producing compensatory eye movements with a very short latency.

Impairment of DVA manifests as oscillopsia—a false sensation of movement of the visual surround during head movements [1, 2]. These symptoms have been well described in bilateral vestibulopathy [3, 4], however, vestibulopathy is not the only cause of DVA impairment. DVA impairment was observed in other diagnoses, such as multiple sclerosis [5]. Vestibular impairment causes decrease of VOR gain but dynamic characteristics of the VOR and adaptive modification by vision are controlled by the cerebellum, mainly the flocculus [6]. In addition, several other mechanisms such as visual tracking, motor preprogramming, prediction, and mental set serve to optimize the compensatory eye to head movements [7]. All of these mechanisms interact synergistically, but there are limitations in their capacity; especially during high frequency, high velocity, or accelerative head movements [7].

Peripheral vestibulopathy has been reported in many CA subtypes, particularly in patients with SCA-3, Friedreich ataxia or cerebellar ataxia with neuropathy and vestibular areflexia syndrome (CANVAS) [8–11]. Thus, it is not surprising that oscillopsia has been reported in patients with degenerative cerebellar ataxias (CA) [9, 12], where it can be the consequence of vestibular impairment, but impaired modulation mechanisms of VOR or other central oculomotor impairment can contribute [12–14]. While oscillopsia is a subjective symptom, DVA can be objectively tested and has been considered as an indirect objective indicator of VOR function. The clinical DVA test provides a bridge between classical, objective diagnostic testing and clinical, subjective observation of functional motor behavior [15].

Although oscillopsia due to the DVA impairment has been demonstrated to be a major factor affecting the quality of life in patients with compensated vestibular lesion [16] and the existence of oscillopsia has been demonstrated also in CA patients [9–11], to our best knowledge no systematic study of DVA has been performed in patients with progressive CA.

The aim of the study was to: 1) describe objective impairment of DVA in patients with CA and vestibular impairment (CA-V) and in patients with CA without vestibular impairment (CA-NV) using clinical DVA test.

2) compare the severity of DVA impairment in CA-V and CA-NV patients with three control groups: a) patients with unilateral and b) bilateral peripheral vestibular lesion and c) healthy age matched controls.

3) estimate the relation of DVA impairment in patients with CA to the presence of peripheral vestibular lesion in these patients and the degree of ataxia expressed by Scale for the assessment and rating of ataxia (SARA) score.

We hypothesized that the DVA would be impaired in both patient groups (CA-V and CA-NV) and this impairment would be proportional mainly to the degree of vestibular impairment and to a lesser extent to the degree of ataxia expressed by SARA score.

## Materials and methods

The study was approved by the Ethical committee of Motol University Hospital. All subjects gave informed consent for participation in the study. Written consent was obtained from each subject.

### Participants

In total, we examined 32 patients with degenerative CA recruited in the Center for hereditary ataxias, Motol University hospital. They were characterized by slowly progressive cerebellar syndrome with insidious onset, brain MRI compatible with degenerative etiology and medical history and laboratory examination without arguments for non degenerative etiology. The CA patients underwent the complete diagnostic protocol including vestibular testing by electronystagmography with calorimetry and video head impulse test (vHIT) (see protocol described below). The presence/absence of spontaneous and gaze nystagmus was determined. According to the results of the caloric testing they were further classified into 2 groups–patients with CA and bilateral vestibulopathy–CA-V (n = 12) and CA without vestibulopathy CA-NV (n = 20). Diagnostic evaluation of patients with cerebellar ataxia included brain MRI, screening for metabolic disorders (vitamin E, free thyroxine and thyroid-stimulating hormone, vitamin B12, folate, alfa-fetoprotein, serum anti-gliadin antibodies, and antiendomysial antibodies), peripheral nerve conduction, and all patients underwent molecular genetic testing for SCA type 1, 2, 3, 6, 7, 8, 12, 17, 28, dentatorubral-pallidoluysian atrophy (DRPLA) and Friedreich ataxia. The severity of ataxia was assessed with SARA scale [17]. Patients with progressive cerebellar syndrome of unknown cause with negative tests for secondary ataxia and negative results of genetic testing were classified as idiopathic late onset cerebellar ataxia (ILOCA). They were clinically and neurophysiologically indistinguishable from hereditary ataxias [18]. Two patients initially diagnosed as ILOCA developed MSA-C. Patients with secondary cause of ataxia were not included in the study. In total, we included 4 SCA-2, 1 SCA-3, 2 SCA-8, 23 ILOCA and 2 MSA-C patients.

We used 3 control groups: patients with bilateral chronic vestibulopathy (BV) (n = 13), patients with unilateral chronic vestibulopathy (UV) (n = 13) and 21 healthy controls (HC). The control groups were age matched to CA patients. Patients with chronic vestibulopathy were recruited from the Neuro-otological centre, Motol University hospital. These patients fulfilled the criteria of unilateral or bilateral caloric hyporeflexia, they were clinically compensated (without spontaneous or gaze nystagmus). Patients with vestibulopathy presented no other neurological impairment, electronystagmography (ENG) proved normal eye tracking, no spontaneous nor gaze nystagmus, normal optokinetic nystagmus and visual suppression of vestibulo-ocular reflex. All vestibular patients underwent brain MRI which showed no signs of cerebellar damage. Etiology of vestibulopathy is listed in Table 1. HC were recruited at the Department of Neurology, Motol University hospital, some of them were patients hospitalized for low back pain, others were partners of the patients with vestibulopathy or cerebellar ataxia, employees of the department or students. They had no history of brain or vestibular disease and denied vertigo or balance problems and were without medication that could influence or induce balance problems. Their neurological examination was normal, the normality of their vestibular function was verified by means of video Head Impulse test. The study was approved by the Ethical committee of Motol University Hospital. All subjects gave informed consent for participation in the study.

**Table 1:** Demographic, clinical characteristics and results.

Demographic characteristics	Cerebellar ataxia		Control groups		
	CA–NV cerebellar ataxia without peripheral vestibulopathy	CA–V cerebellar ataxia with peripheral vestibulopathy	BV bilateral vestibulopathy	UV unilateral vestibulopathy	HC healthy controls
Number of subjects	20	12	13	13	21
Age M (SD)	56.65 (15.43)	67.5 (16.13)	59.07 (19.22)	52.15 (10.15)	52.57 (20.46)
Etiology	4 SCA-2	1 SCA-3	3 ototoxicity	10 vestibular neuronitis 2 morbus Meniere 1 vascular	
	2 SCA-8	11 ILOCA	10 idiopathic		
	2 MSA-C				
	12 ILOCA				
Disease duration (in years) M (SD)	7.65 (5.63)	7.16 (4.58)	5.05 (6.59)	1.94 (2.32)	
SARA M (SD)	11.32 (6.94)	11.22 (4.72)	NA	NA	NA
Caloric irrigation (SCV °/s) age adjusted score M (SE)	26.79 (23.98–29.58)	9.71 (6.26–13.15)	5.67 (2.34–8.99)	15.46 (12.2–18.72)	NA
VOR gain of horizontal SCC * age adjusted score M (SE)	1.05 (0.95–1.14)	0.71 (0.58–0.83)	0.61 (0.48–0.72)	0.86 (0.73–0.97)	1.09 (0.99–1.18)
VOR gain of vertical SCC * age adjusted score M (SE)	0.82 (0.74–0.89)	0.62 (0.51–0.71)	0.63 (0.53–0.72)	0.79 (0.69–0.88)	0.92 (0.84–1.00)
DVA score at 1Hz (in chart lines) age adjusted score M (SE)	1.5 (0.88–2.11)	2.32 (1.49–3.13)	2.44 (1.67–3.20)	1.04 (0.27–1.81)	0.23 (-0.37–0.83)
DVA score at 2Hz (in chart lines) age adjusted score M (SE)	3.4 (2.65–4.15)	4.13 (3.13–5.12)	5.51 (4.58–6.44)	1.90 (0.96–2.83)	0.57 (-0.16–1.31)
Subjectively perceived oscillopsia (% of subjects)**	55%	58.3%	76.9%	53.8%	0%

M–mean, SD–standard deviation, SE–standard errors (lower- and upper-95 percent confidence limits), SARA—scale for the assessment and rating of ataxia, DVA–dynamic visual acuity, SCV–nystagmus slow-component velocity response, VOR–vestibulo-ocular reflex, NA–not available.

* values of VOR gains are mains from right and left side. We have decided to report these values as a mean from both sides as the DVA test does not allow to recognize right and left movements.

** positive response to the question “Have you ever had problems with wobbling, jumping or blurring of vision?”

### Vestibular function tests

#### Video Head Impulse test (vHIT)

All subjects in our study underwent video Head Impulse test (vHIT). We used a vHIT system ICS Impulse, GN Otometrics, Denmark. It is a lightweight video-oculography (VOG) system with built-in 9-axis motion tracking sensor and a high-speed digital camera that captures the image of the right eye. The device software (OTOsuite Vestibular Software Version 1 I 20 Build 310) automatically evaluates individual vHIT and calculates mean VOR gains.

Head impulses were performed by specially trained examiners. Patients were seated and instructed to fixate a target at reference position straight ahead at a distance of 100cm. Vestibulo-ocular reflex was evoked by passive head impulses in the plane of a pair of semicircular canals (SCC) with a complementary opposite optimal sensitivity, directed toward either side in random sequence. Head velocity was up to 100–200°/sec, the amplitude of the impulse varied from 5°to 20° [19–21]. Head impulses with comparable time course were recorded for VOR gain evaluation, inadequate vHIT trials (wrong velocity or stimulation plain) were rejected automatically by a software filtering algorithm.

All six semicircular canals were examined, each with 15–20 impulses. VOR gain was calculated as the ratio of the area under eye velocity curve and the area under the head velocity curve during the period from the start to the end of the head impulse [22]. Average gain and asymmetry index were calculated from the gain values of individual head impulses. The vHIT results were considered as pathological if the averaged gain of all impulses for the respective canal was less than age-dependent normative data (typically 0.7–0.8 for horizontal canal) and overt and covert correcting saccades were recorded [22].

#### Electronystagmography

In addition, patients with CA and vestibular patients underwent ENG performed with four-channel equipment (Toennies Nystagliner, Germany). ENG recording included calibration of ocular movements, detection of spontaneous and gaze nystagmus (°), eye tracking test (qualitative irregularity), optokinetic nystagmus test (gain, symmetry, and regularity), saccade test (velocity, latency), rotatory test without and with gaze fixation and caloric test. Bithermal caloric testing consists of water irrigation at 30°C and 44°C according to the method Fitzgerald-Hallpike [23]. The presence of peripheral vestibulopathy was based on caloric irrigation test and rotational hyporeflexia. Criteria for vestibulopathy according to caloric irrigation were the sum of the maximal peak velocities of the slow phase caloric-induced nystagmus for stimulation with warm and cold water <15°/s. The results of caloric irrigation were expressed as the mean nystagmus slow-component velocity response (SCV) from the right and the left ear. One patient from BV group could not undergo caloric irrigation and the bilateral vestibulopathy was defined by severe rotational hyporeflexia with VOR gain 0.01, where the gain is defined as a peak slow-phase eye velocity divided by the peak stimulus (chair) velocity [24] and further was confirmed by low bilateral VOR gain in vHIT.

#### Clinical Dynamic Visual Acuity (DVA) test

Based on the previous studies, the clinical DVA test was done with passive head movement to minimize the effect of central preprogramming on gaze control [25–27]. We used LCD optotype NIDEK SC-1600 that displays five letters in a single line with corresponding logMAR (logarithm of the Minimum Angle of Resolution), with a change in acuity of 0.1 logMAR between each line. The letters of equal legibility were displayed in random sequence to avoid memorization. The subjects were seated in a chair 5m from the monitor and ask to identify letters, beginning with line 0.1 (decimal value), continuing for successive lines until the subject missed three of the five optotypes on a line. The logMAR values of this line (“missed three” score) and that of the lowest line on which all optotypes were correct were recorded. This was done under the following conditions: head stationary (static visual acuity–SVA) and head passively rotated sinusoidally 20° from center to the left and right (dynamic visual acuity—DVA) to the beat of a metronome at 1Hz and 2 Hz (previously recommended parameters known to challenge the VOR) [25–32]. The 2Hz has been considered as a more challenging condition. The amplitude of a head movement was controlled by the use of accelerometer and gyroscope sensor WMS 3.0, Princip a.s., Prague, providing feedback for the examiner to achieve accurate head movements. A dynamic visual acuity score (DVA score) was calculated as a difference between SVA and DVA, in chart lines on the logMAR chart [30]. The principle of this test does not allow to evaluate differences between right and left head rotation. According to Rine et al., we defined the decline of DVA ≥2S.D. above the normative mean as abnormal [32], in our case, it represents DVA score higher than one.

In addition to the objective measurement of the clinical DVA test, the presence of oscillopsia was estimated using the question “Have you ever had problems with wobbling, jumping or blurring of vision?”.

### Statistics

Descriptive statistics are provided by diagnosis with age-adjusted means for DVA1Hz, DVA2Hz, mean VOR gain of the horizontal semicircular canals in vHIT and the SCV—calorimetry. In order to compare age-adjusted means, analysis of variance (ANOVA) and post-hoc tests were carried out with a Tukey correction for increased Type I error.

In order to examine the effect of VOR and SARA Score on DVA in ataxia patients, we fit several linear regression models. The first model examined the effect of VOR gain of horizontal semicircular canals (SCC) on DVA after controlling for age. The second model examined the effect of SARA score on DVA after controlling for age. The third model examined the effects of SARA score and VOR Gain of horizontal SCC on DVA after controlling for age. For each model, adjusted-R^2^ values are reported, which estimate the amount of variance in the DVA score that was explained by the models.

## Results

### Demographic and clinical characteristics

Basic demographic and clinical characteristics of the groups are listed in Table 1. There was no significant difference in age between groups (p = 0.13). No significant difference was found in SARA score between CA-V and CA-NV patients (p = 0.96). DVA decrease was detected in 84.3% of CA patients and in 79.9% of patient with vestibulopathy. The presence of oscillopsia using screening question was reported by both subgroups of patients with CA as well as both vestibular control groups but not by healthy control (56.2% of CA: 58.3% of CA-V and 55% of CA-NV patients reported the presence of oscillopsia compared to 53.8% UV, 76.9% BV and 0% HC).

#### Ocular motor abnormalities in CA

According to ENG testing, 5 patients had spontaneous nystagmus– 3 in group CA-NV (2 of them with downbeat nystagmus) and 2 in group CA-V. Gaze nystagmus was observed in 50 percent of patients–in 9 patients with CA-NV (2 of them with downbeat nystagmus) and in 7 patients with CA-V (2 of them with downbeat nystagmus).

### Vestibulo-ocular reflex

As expected, the VOR gain of horizontal semicircular canals assessed by vHIT test differed significantly between CA-NV and CA-V (p = 0.002). The impairment of VOR in CA-V patients was similar to patients with BV. The values of VOR gain in CA-NV as well as in HC were within the normal range.

### Dynamic visual acuity

**Fig 1:**
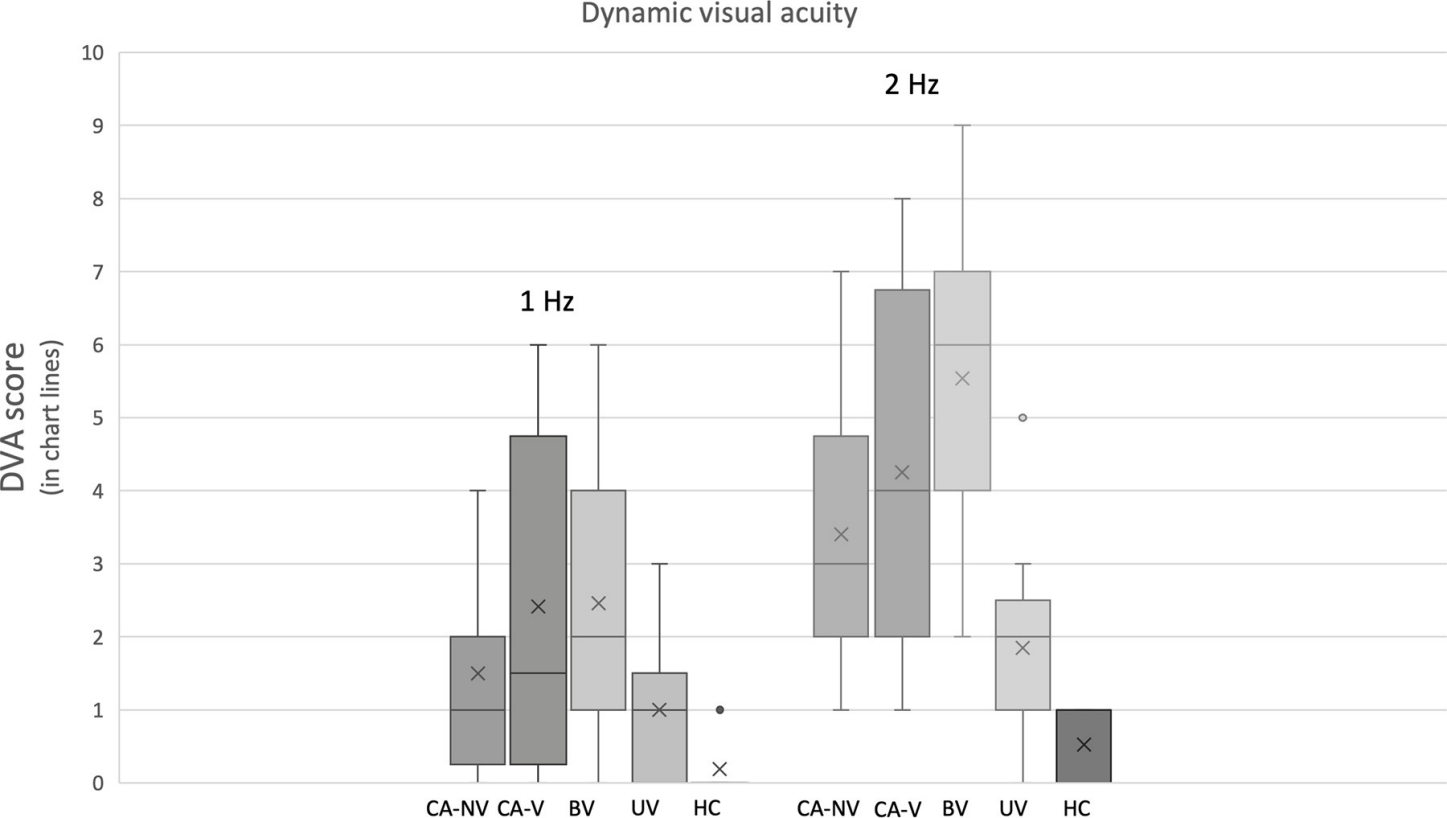
Distribution of DVA scores among the groups (boxplot graph). CA-NV–cerebellar ataxia without vestibulopathy, CA-V–cerebellar ataxia with vestibulopathy, BV–bilateral vestibulopathy, UV—unilateral vestibulopathy, HC–healthy controls.

In total 91.6% of CA-V and 80% of CA-NV had pathological DVA score (scored more than 1) at 2Hz and 50% of CA-V and 40% of CA-NV at 1Hz compared to 100% of BV, 53.8% of UV and 0% of HC at 2Hz and 69.2% of BV, 23% of UV and 0% of HC at 1Hz. The distribution of DVA scores among the groups are illustrated in Fig 1.

Both CA-V and CA-NV scored worse compared to healthy controls at both 1 and 2Hz condition (p<0.001) but did not differ from each other. In addition, CA-V patients scored worse compared to UV at 2Hz, but they did not differ from BV at either 1 or 2Hz and CA-NV patients scored better than BV at 2Hz. These results are summed in Table 2.

**Table 2:** Comparison between groups.

**DVA score (in chart lines) at 2Hz: *p* < .001**					
	CA-NV	CA-V	Bilateral vestibulopathy	Unilateral vestibulopathy	Healthy controls
**Age-Adjusted Score**	**3.40**	**4.13**	**5.51**	**1.90**	**0.57**
CA-NV	-				
CA-V	NS	-			
Bilateral vestibulopathy	.006**	NS	-		
Unilateral vestibulopathy	NS	.02*	< .001***	-	
Healthy controls	< .001***	< .001***	< .001***	NS	-
**DVA score (in chart lines) at 1Hz: *p* < .001**					
	CA-NV	CA-V	Bilateral vestibulopathy	Unilateral vestibulopathy	Healthy controls
**Age-Adjusted Score**	**1.50**	**2.32**	**2.44**	**1.04**	**0.23**
CA-NV	-				
CA-V	NS	-			
Bilateral vestibulopathy	NS	NS	-		
Unilateral vestibulopathy	NS	NS	NS	-	
Healthy controls	.03*	.001**	< .001***	NS	-
**Mean VOR gain of horizontal SCC: *p* < .001**					
	CA-NV	CA-V	Bilateral vestibulopathy	Unilateral vestibulopathy	Healthy controls
**Age-Adjusted Score**	**1.05**	**0.71**	**0.61**	**0.86**	**1.09**
CA-NV	-				
CA-V	< .001***	-			
Bilateral vestibulopathy	< .001***	.75	-		
Unilateral vestibulopathy	.10	.48	.03*	-	
Healthy controls	.98	< .001***	< .001***	.03*	-
**Mean VOR gain of vertical SCC: *p* < .001**					
	CA-NV	CA-V	Bilateral vestibulopathy	Unilateral vestibulopathy	Healthy controls
**Age-Adjusted Score**	**0.82**	**0.62**	**0.63**	**0.79**	**0.92**
CA-NV	-				
CA-V	.02*	-			
Bilateral vestibulopathy	.03*	.99	-		
Unilateral vestibulopathy	.99	.11	.14	-	
Healthy controls	.29	< .001***	< .001***	.21	-

p values are reported for significant differences.

Abbreviations: CA-NV—cerebellar ataxia without peripheral vestibulopathy, CA-V—cerebellar ataxia with peripheral vestibulopathy, DVA–dynamic visual acuity, VOR–vestibulo-ocular reflex, NS–non-significant.

### Relation of DVA to vestibular tests or SARA

Among cerebellar ataxia participants, a one-unit increase in mean VOR gain of horizontal SCCs in vHIT was associated with -2.69 lower units on DVA 1Hz and –2.43 lower units on DVA 2Hz after controlling for the effect of age; among control participants, a one-unit increase in mean VOR gain was associated with -2.96 lower units on DVA 1Hz and -7.17 lower units on DVA2Hz after controlling for the effect of age (all ps < .001). There was no significant relation between SCV in caloric irrigation and DVA in any group (all ps>.1).

When examining the effect of SARA score on DVA in ataxia patients, we fit several linear regression models. The amount of variance in the DVA score after controlling for age that was explained by the models was expressed as Adjusted-R^2^. The first model examining the effect of VOR gain of horizontal SCCs on DVA 1 and DVA 2Hz explained 16.6% and 8.7% of variance respectively. The second model examining the effect of SARA Score on DVA 1 and 2Hz explained 4.1% and 3.7% of variance respectively. The third model examining combined effects of SARA Score and VOR gain on DVA 1 and 2Hz explained 24.4% and 14.2% of variance respectively.

## Discussion

We have demonstrated high prevalence of dynamic visual acuity impairment in both groups of patients with cerebellar ataxia (CA-NV and CA-V) in contrast to healthy controls. CA-V patients showed similar impairment of DVA as patients with bilateral vestibulopathy. More than a half of CA patients reported signs of oscillopsia. Objective DVA impairment in CA patients was related to the vestibular function measured by video head impulse test (VOR gain) and to a lesser extend to SARA score. The best predictive value was achieved by calculating VOR gain and SARA scores together.

To our best knowledge, we are the first who objectified the DVA impairment in patients with progressive cerebellar ataxia. 37% of our patients with CA showed signs of peripheral vestibulopathy and the severity of DVA impairment in this group was comparable to patients with bilateral vestibulopathy.

On the other hand, a significant decrease of DVA was highly prevalent also in the group of patients with cerebellar ataxia without vestibulopathy. These results bring evidence that DVA impairment in CA is probably also related to other factors, such as impaired modulation of vestibulo-ocular reflex [6, 13, 14] by the flocculus. The flocculus has been proposed to be a center of learning in VOR gain–it is a part of the accessory path for the major VOR arc and through visual feedback, the flocculus rapidly corrects the VOR in order to maintain the constancy of retinal images [33].

Cerebellar damage can lead to oculomotor deficits, such as spontaneous or gaze nystagmus, impaired smooth pursuit, dysmetric saccades and misalignment of the eyes [34, 35] that could cause greater retinal slip even when the VOR is intact and affect many visual perceptual tasks. We assume that larger difference between CA and vestibulopathy patients would be seen during active DVA test (with head movements initiated voluntarily by the patient). We expect that the patients with CA would score worse than the patients with vestibulopathy due to their impaired central preprogramming of eye movements during predictable head movements [25].

In total, oscillopsia was reported in 56.2% of our patients with cerebellar ataxia. In previous studies, the presence of oscillopsia was reported in part of patients with Friedreich ataxia [11], SCA-3 [10] and other spinocerebellar ataxias [9], although the prevalence was lower. Zeigelboim et al. examined 43 patients with SCA (SCA 2,3,4,6,7,10 and ILOCA) and described blurred vision in 11.6% [9], but they did not specify if the blurred vision was movement related. Fahey et al. reported complaints of oscillopsia or blurred vision in 4 of 15 patients with Friedreich ataxia [11]. Gordon et al. tested seven patients with SCA3 and only one reported oscillopsia during locomotion [10]. The differences in the prevalence of subjective oscillopsia between the studies could be caused by a relatively small number of patients or different adaptation to oscillopsia in these groups of patients. Our cohort of patients with CA was rather heterogenous, including patients with different types of degenerative CA in different stages of the disease.

DVA decrease was detected in 84.3% of CA patients but only 56.2%, reported oscillopsia while patients with vestibulopathy complained about oscillopsia in 65.3% and pathological clinical DVA test had 76.9% of these patients. Different adaptation to oscillopsia could explain disparate degrees of oscillopsia and DVA impairment in CA. In patients with vestibulopathy, it is partly related to the patient’s attitude to the recovery process and partly to the development of tolerance to the movement of images on the retina during self-motion [36] and downregulation of the early visual cortex excitability [37]. Oscillopsia is not a frequent symptom in patients with oculomotor disorders, nor in patients with congenital nystagmus, probably due to the reduction of visual motion sensibility [38]. This might be a reason of weaker perception of oscillopsia also in CA patients.

Severity of DVA impairment was significantly related to the gain of vestibulo-ocular reflex evaluated by vHIT but not to the caloric irrigation (SCV). This is in agreement with widely recognized fact that although caloric irrigation has been used as a standard test of vestibular function, it should be viewed as non-physiological stimulation. In contrary, the vHIT could be considered as more ecologically valid and thus more suitable for detecting residual vestibular function [39].

When analyzing the effect of SARA score on DVA in ataxia patients, we found only weak contribution of SARA to the regression model, showing that the contribution of the severity of ataxia on DVA is marginal compared to vestibular dysfunction and the presence of DVA impairment is rather independent from severity of ataxia in CA patients.

It has been demonstrated previously that the DVA impairment in patients with BV decreases the quality of life and interact significantly with activities of daily living and causes the working disability [16]. The DVA impairment in these patients may cause difficulties particularly in motion-intense activities including sports or car driving [40]. Although it is difficult to separate different factors affecting the quality of life in CA patients, the same prevalence and severity of DVA impairment in CA-V group as in BV means that DVA impairment could significantly contribute to the disability and impairment of quality of life also in CA patients.

Our study has several limitations: we used a heterogenous population of patients with genetically defined CA and ILOCA, so the study does not allow to provide the direct link to the pathophysiology of DVA impairment in the whole sample. On the other hand, the method of recruiting the real clinical population from the Center of hereditary ataxias allows generalization to the routine clinical praxis.

Another limitation is, that we performed only passive clinical DVA test in a horizontal plane. To effectuate the examination in both horizontal and vertical plane and in active and passive way would be of great interest. It is possible that passive performance of clinical DVA test provides a purer examination of VOR, however, actively performed DVA test would be closer to natural behavior.

## Conclusion

To our best knowledge, this is the first systematic study of DVA in patients with progressive cerebellar ataxia. The impairment of DVA is very common in CA patients, which corresponds well with the high prevalence of subjectively reported oscillopsia. The DVA impairment is closely related to the degree of vestibular dysfunction in CA patients, relies only marginally on the degree of ataxia but has a high prevalence even in patients with CA without vestibular lesion, proving that both visual and vestibular compensation of the retinal slip contributes to dynamic visual acuity. Peripheral vestibulopathy as well as the central mechanisms may play important role in the pathophysiology of DVA impairment in these participants.

We suggest that clinical DVA tests should be a standard part of clinical evaluation in CA patients as this information can help to target vestibular and oculomotor rehabilitation. Further studies are needed to evaluate the impact of DVA impairment and oscillopsia on quality of life in CA patients. Future studies should also focus to compare active and passive DVA in CA patients.

## Supporting information

S1 Dataset Minimal dataset. (XLSX)
